# Replicase-based plasmid DNA shows anti-tumor activity

**DOI:** 10.1186/1471-2407-11-110

**Published:** 2011-03-28

**Authors:** B Leticia Rodriguez, Zhen Yu, Woon-Gye Chung, Richard Weiss, Zhengrong Cui

**Affiliations:** 1 Pharmaceutics Division, College of Pharmacy, University of Texas, Austin, TX 78712, USA; 2 Department of Pharmaceutical Sciences, College of Pharmacy, Oregon State University, Corvallis, OR 97331, USA; 3 Division of Allergy and Immunology, Department of Molecular Biology, University of Salzburg, Hellbrunnerstrasse 34, 5020 Salzburg, Austria

## Abstract

**Background:**

Double stranded RNA (dsRNA) has multiple anti-tumor mechanisms. Over the past several decades, there have been numerous attempts to utilize synthetic dsRNA to control tumor growth in animal models and clinical trials. Recently, it became clear that intracellular dsRNA is more effective than extracellular dsRNA on promoting apoptosis and orchestrating adaptive immune responses. To overcome the difficulty in delivering a large dose of synthetic dsRNA into tumors, we propose to deliver a RNA replicase-based plasmid DNA, hypothesizing that the dsRNA generated by the replicase-based plasmid in tumor cells will inhibit tumor growth.

**Methods:**

The anti-tumor activity of a plasmid (pSIN-β) that encodes the sindbis RNA replicase genes (nsp1-4) was evaluated in mice with model tumors (TC-1 lung cancer cells or B16 melanoma cells) and compared to a traditional pCMV-β plasmid.

**Results:**

In cell culture, transfection of tumor cells with pSIN-β generated dsRNA. In mice with model tumors, pSIN-β more effectively delayed tumor growth than pCMV-β, and in some cases, eradicated the tumors.

**Conclusion:**

RNA replicase-based plasmid may be exploited to generate intracellular dsRNA to control tumor growth.

## Background

Double stranded RNA has multiple anti-tumor mechanisms that may be potentially exploited to control tumor growth. It is known to be pro-apoptotic, anti-proliferative, and anti-angiogenic [1-3]. It is also a potent inducer of type I interferons (IFN-α/β) [2,4], which are pro-apoptotic and immuno-stimulatory as well [1,2,5]. Intracellular dsRNA can activate various pathways, including anti-proliferative dsRNA dependent protein kinase (PKR), IFN inducible 2'-5'-adenylate synthetase/Rnase L system, and oligo A synthetase [4,6,7], which can lead to apoptosis. Intracellular dsRNA is recognized primarily by retinoic acid-inducible gene I (RIG-1) and melanoma differentiation-associated gene 5 (Mda5) [8-10]. Extracellular dsRNA recognition occurs by Toll-like receptor (TLR3) membrane bound receptor [8,11].

Over the past several decades, there had been numerous attempts to utilize synthetic dsRNA such as polyriboinosinic-polyribocytidylic acid, poly (I:C), to control tumors in animal models and clinical trials [3,12-14]. In general, it was found that synthetic dsRNA only slightly delayed tumor growth [15-17]. Increasing the dose of the synthetic dsRNA to improve its anti-tumor activity is not feasible because of the dose-dependent severe adverse effects [15,18]. Recently, there is a reviving interest in exploiting the anti-tumor activity of synthetic dsRNA by improving the delivery of dsRNA into tumor cells [19]. For example, Shir *et al*. (2006) reported the total regression of implanted human breast cancers or glioblastoma in mouse models when poly (I:C) was intratumorally injected and targeted into the tumor cells using epidermal growth factor as a ligand [19]. Using B16-F10 melanoma in a mouse model, Fujimura *et al*. (2006) reported the elicitation of tumor-specific CD8^+ ^T lymphocyte responses by peritumoral injection of poly (I:C) [3]. Others have exploited the immuno-stimulatory activity of dsRNA by immunizing with tumor cells with intracellular synthetic dsRNA [20]. It became clear that intracellular dsRNA was more effective than extracellular dsRNA in promoting tumor cells to undergo apoptosis and orchestrating the initiation of adaptive immune responses [20-22].

Sindbis virus is an alpha virus that contains a single positive stranded RNA encoding its own RNA replicase [23,24]. An anti-sense RNA is transcribed, and it functions as a template for the synthesis of sense RNA. RNA-dependent RNA polymerase activity was found on the nonstructural protein (nsP4) [25,26]. Sindbis viral vectors deficient in replication genes have been shown to efficiently target and kill tumor cells *in vivo *[27-29]. However, concerns regarding uncontrolled vector propagation and toxicity suggest that non-viral based plasmids may offer a safer alternative [30]. Previously, the replicase genes (nsp1-4) from sindbis virus have been cloned into a plasmid and placed under the control of cytomegalovirus (CMV) promoter [23]. When transfected into cells, the replicase genes are expressed, and the resultant replicase complex allowed the formation of intracellular dsRNA [23,31]. Therefore, we sought to deliver the replicase-based plasmid into tumor cells, hypothesizing that the RNA replicase based plasmid will generate dsRNA inside tumor cells and inhibit the tumor growth. This strategy is advantageous because it would avoid the delivery of a large dose of synthetic dsRNA *in vivo*, which is rather challenging; while there have been cases of successful delivery of DNA into tumor cells [30,32,33]. Another advantage of utilizing plasmid DNA is that the unmethylated CpG motifs on the plasmid are also immuno-stimulatory [34,35]. CpG motifs were shown to have anti-tumor activity by activating natural killer cells and by inducing the secretion of cytokines such as IL-6, TNF-α, and IFN-γ [34].

In the present study, a sindbis replicase-based plasmid pSIN-β was used. In the plasmid, the sindbis nsp1-4 genes were under the control of a CMV promoter [23]. Using a model mouse lung cancer cell line, TC-1, it was shown that when transfected into cells in culture, the pSIN-β generated dsRNA, and the resultant dsRNA seemed to be pro-apoptotic. In mouse model, the pSIN-β significantly inhibited the growth of the TC-1 tumors. Similar anti-tumor activity was also observed when the pSIN-β was used to treat B16 melanoma in mice.

## Methods

### Plasmids

Plasmid pCMV-β was from the American Type Culture Collection (ATCC, Manassas, VA). The pSIN-β plasmid was constructed following a previously described method [23]. The pSIN-β-Δnsp was constructed in two steps. First, the pSIN-β was digested with *Pst I *(Invitrogen, Carlsbad, CA), and the resultant fragment was gel extracted and purified using a PureLink Gel Extraction kit (Invitrogen). The DNA fragment was further digested with *Hind III *(Invitrogen). The correct fragment was gel extracted, and the adhesive ends were ligated using T4 DNA ligase (Invitrogen). All plasmids were amplified in *E. coli *DH5α under selective growth conditions.

Plasmid DNA was methylated at CpG sites with CpG methyl transferase (M.SssI) (New England BioLabs, Beverly, MA). The M.SssI methylates at the carbon position 5 of cytosine residues within double stranded recognition sequence. Methylation reaction containing 2 U of methylase per μg of DNA was incubated at 37°C for at least 3 h. The extent of methylation by the M.SssI was determined using a BstU I endonuclease assay (Invitrogen). Plasmid was purified from bacteria using a QIAGEN midiprep kit (Valencia, CA). Large scale plasmid preparation was performed by GenScript (Piscataway, NJ).

### Cell lines and culture

Mouse lung tumor cells (TC-1, ATCC, CRL-2785) and mouse melanoma cells (B16-F10, ATCC, CRL-6475) were cultured in RPMI 1640 medium (Invitrogen) and DMEM medium (Invitrogen), respectively. The media were supplemented with 10% fetal bovine serum (FBS, Invitrogen), 100 U/ml of penicillin (Invitrogen), and 100 μg/ml of streptomycin (Invitrogen).

The ovalbumin (OVA)-expressing B16-OVA cell line was generously provided by Dr. Edith M. Lord and Dr. John Frelinger (University of Rochester Medical Center, Rochester, NY) [36]. B16-OVA cells were cultured in RPMI 1640 medium supplemented with 5% FBS and 400 μg/ml of G418 (Sigma).

### *In vitro *Transfection

TC-1 cells were seeded in 24 or 48 well plates (20 000 cells/well) and incubated at 37°C, 5% CO_2 _for 24 h or until 60% confluency followed by transfection using plasmid DNA (0.15 or 0.40 μg as where mentioned) complexed with Lipofectamine^® ^(Invitrogen) following the manufacturer's instruction. The transfection medium was replaced with fresh medium 3 h later.

### Semi quantitative RT-PCR

Total RNA was isolated from TC-1 cells (1 × 10^7^) transfected with plasmid using a QIAGEN RNeasy mini kit. On-column DNase digestion was performed using RNase-free DNase set (QIAGEN) to eliminate DNA contamination. The RNA quality was assessed using the OD260/OD280 ratio.

Reverse transcriptase reaction was performed using Invitrogen SuperScript III™kits (Cat No. 11752-050 or No. 18080-093) with oligo dT primers or sindbis nsp4 gene specific primers (nsp4-1, p4F (5'-CCGGAATGTTCCTCACACTT-3') and p4R (5'-GGAATGCTTTTGCTCTGG-3')). Polymerase chain reaction was completed utilizing cDNA from the reverse transcription and primer set p4F/p4R, which amplified a 501 base pair fragment of the nsp4 gene. Reactions were conducted using an Eppendorf Mastercycler (Hauppauge, NY) for 30 cycles: 94°C for 5 min, 94°C for 30 s, 55°C for 30 s, 72°C for 30 s, and a 5 min final extension at 72°C. The nsp4 gene fragment was amplified using platinum *taq *DNA polymerase (Invitrogen). The PCR products (25 μl) were analyzed using agarose gel electrophoresis.

### Enzyme-linked immunosorbent assay (ELISA)

The presence of dsRNA in TC-1 cells (n = 3) transfected with the plasmid was confirmed using ELISA as previously described with modification [37]. Briefly, 96-well plates were coated at 4°C overnight with 1 μg total RNA diluted in PBS. Plates were washed with PBS/Tween 20 (10 mM, pH 7.4, 0.05% Tween 20, Sigma-Aldrich, St. Louis, MO) and blocked with 4% (w/v) bovine serum albumin (BSA, Sigma-Aldrich) in PBS/Tween 20 for 1 h at 37°C. Plates were washed again with PBS/Tween 20. Monoclonal anti-dsRNA antibody J2 (English & Scientific Consulting Bt. Szirák, Hungary) was added to each well following the removal of the blocking solution. The plates were incubated for an additional 3 h at 37°C. Horseradish peroxidase (HRP) labeled goat anti-mouse IgG2a (5 000-fold dilution Southern Biotechnology Associates, Birmingham, AL) was added to the wells, followed by 1 h of incubation at 37°C. The presence of bound secondary antibody was detected after a 30 min incubation with 3,3',5,5'-tetramethylbenzidine substrate (TMB) (Sigma-Aldrich). The reaction was stopped by the addition of sulfuric acid (0.2 M, Sigma).

### Determination of cell viability

The number of viable TC-1 cells was determined using a 3-(4,5-dimethylthiazol)-2-,5-diphenyltetrazolium bromide (MTT) kit (Sigma-Aldrich) 24, 48, and 72 h after the initiation of the transfection (n = 4) [12]. Cells treated with sterile PBS were used as a control. Formula used to calculate the relative cell number (%) was: Relative cell number = 100 × number of live cells transfected with pCMV-β (pSIN-β, or pSIN-β-Δnsp)/number of live cells treated with sterile PBS.

### Preparation of plasmid DNA-liposome lipoplexes

Cationic liposomes were prepared using cholesterol (Sigma-Aldrich), egg phosphatidylcholine (Avanti Polar Lipids, Inc, Alabaster, AL), and 1,2,-dioleoyl-3-trimethylamonium-propane (DOTAP, Avanti) at a molar ratio of 4.6:10.8:12.9 by thin film hydration method followed by membrane extrusion (1, 0.4, and 0.1 μm, sequentially) [38]. The final concentration of DOTAP in the liposome was 10 mg/ml. The plasmid-liposome lipoplexes were prepared by mixing equal volumes of plasmid DNA (25 μg in 25 μl) solution and liposome suspension containing 50 μg of DOTAP liposomes. The mixture was allowed to stay at room temperature for at least 15 min before further use. Particle size was measured using a Malvern Zetasizer Nano ZS (Worcestershire, United Kingdom). The size of the liposomes was 110 ± 0.6 nm with a polydispersity index (PI) of 0.121. The pSIN-β-liposome lipoplexes were 255 ± 31 nm (PI, 0.177). The pCMV-β-liposome lipoplexes were 249 ± 33 nm (PI, 0.183). The sizes of the two lipoplexes were not statistically different (p = 0.83, t-test, n = 3).

### Animal studies

All animal studies were carried out following the National Institutes of Health animal use and care guidelines. Animal protocol was approved by the Institutional Animal Care and Use Committee at the University of Texas at Austin. Female C57BL/6 mice (6-8 weeks) were from Simonsen Laboratories (Gilroy, CA) or Charles River laboratories, Inc. (Wilmington, MA). Female athymic nude mice (6-8 weeks) were from Charles River laboratories. Mice were subcutaneously injected with TC-1, B16/F10, or B16-OVA cells (5 × 10^5^) in the right flank. When tumors reached an average diameter of 3-4 mm, the plasmid DNA-liposome lipoplexes were injected subcutaneously peritumorally (s.c., p.t.) for 5 or 10 consecutive days [12,15,19]. The dose of the plasmid DNA was 25 μg DNA per mouse per injection. Tumor size was measured using a digital caliper and calculated using the following equation [39]: tumor diameter = (Length + Width)/2. To examine whether the nsp genes were expressed *in vivo*, pCMV-β, pSIN-β, or pSIN-β-Δnsp (25 μg) was injected into the gastrocnemius muscles in the hind legs of mice (n = 2). After 24 h, the injected muscle tissues were collected and homogenized using TRIzol reagent (Invitrogen) to isolate total RNA. RT-PCR was performed to amplify nsp4 gene or β-gal gene using the nsp4-1 primers or the β-gal primers (5'-GACGTCTCGTTGCTGCATAA-3'; 5'-CAGCAGCAGACCATTTTCAA-3').

### Histology

TC-1 tumors in mice that were treated for 6 consecutive days with plasmids were collected, fixed in formaldehyde, embedded in paraffin, and sectioned. Immunohistochemistry was performed to detect apoptosis using the anti-ACTIVE caspase-3 antibody (Promega, Madison, WI) according to manufacturer protocol. Fifteen random fields per sample at 40 × magnification were scored for cleaved caspase-3. Apoptotic index was determined based on the % of cleaved caspase-3 positive cells found within total cells counted [12].

### Quantification of IFN-α in mouse serum samples

Mice were subcutaneously injected with 125 μg of plasmid DNA in lipoplexes (DNA/liposomes, 1:2, w/w). Ten h later, serum was collected, and the concentration of IFN-α was determined using a mouse IFN-α (Mu-IFN-α) ELISA kit (PBL Biomedical Laboratories, Piscataway, NJ).

### Statistical analysis

Statistical analyses were completed using ANOVA followed by Fisher's protected least significant difference procedure. A p-value of < 0.05 (2-tail) was considered statistically significant.

## Results and discussion

### Generation of dsRNA by transfecting pSIN-β into tumor cells

**Figure 1:**
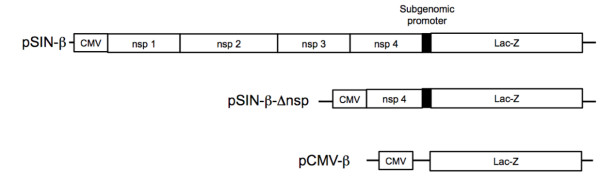
**A schematic of plasmids used in this study**. CMV, cytomegalovirus promoter; Lac-Z, β-galactosidase; nsp, sindbis virus sequences coding for the nonstructural proteins (nsp1-4). pSIN-β-Δnsp (8,727 bp), pSIN-β (14,869 bp), pCMV-β (7,164 bp).

**Figure 2:**
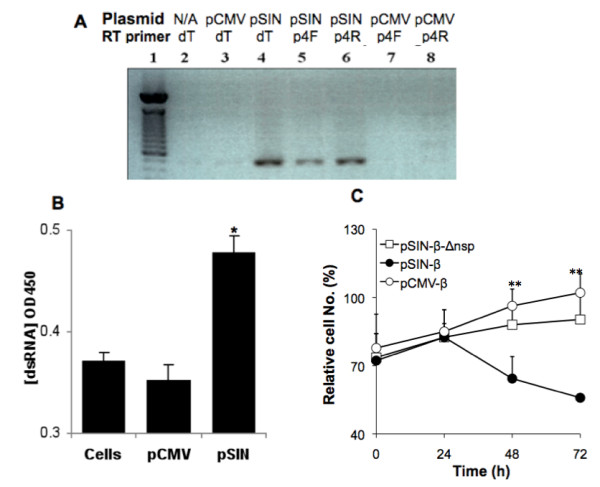
**Generation of dsRNA in tumor cells transfected with pSIN-β**. **(A)**. RT-PCR confirmed the presence of sindbis virus nsp4 gene mRNA and its anti-sense strand in tumor cells transfected with pSIN-β. TC-1 cells were transfected with pCMV-β (pCMV) or pSin-β (pSIN), or left untreated (N/A). Total RNA was reverse transcribed into DNA with oligo dT primer or primers specific to the nsp4 gene (forward p4F or reverse p4R) before PCR amplification. This experiment was repeated twice with similar results. **(B)**. ELISA confirmed the presence of an elevated level of dsRNA in TC-1 cells transfected with pSIN-β (n = 3). Total dsRNA was isolated from TC-1 cells transfected with pCMV-β or pSIN-β and used to coat ELISA plate. The primary Ab was the J2 anti-dsRNA IgG2a. *, p = 0.004. **(C)**. Transfection of pSIN-β into TC-1 cells inhibited cell growth. TC-1 cells (20 000 cells/well) were transfected with the same amount (0.4 μg) of pCMV-β, pSIN-β, or pSIN-β-Δnsp (n = 4). Cell numbers were quantified using MTT assay and normalized to cells treated with sterile PBS. Data shown are mean ± S.E.M. **, at 48 and 72 h, the value of the pSIN-β were different from that of the pCMV-β and the pSIN-β-Δnsp (p < 0.05).

Shown in Figure 1 are maps of the plasmids used. In the pCMV-β, the β-galactosidase gene is driven by the CMV promoter. In the pSIN-β, the nsp1-4 genes are driven by a CMV promoter, while the β-galactosidase gene is driven by a sindbis viral subgenomic promoter. The pSIN-β-Δnsp was constructed by deleting the nsp1-3 and part of the nsp4 genes from the pSIN-β. To confirm that the pSIN-β plasmid can produce dsRNA when delivered into cells, TC-1 cells were transfected with pSIN-β, and the total RNA was extracted from the cells 24 h later. The total RNA was reverse transcribed with either oligo dT primers or nsp4 gene specific primers (p4-F or p4-R). The cDNA was then amplified with nsp4-specific primers. A 501 bp nsp4 gene fragment was observed in all samples transfected with pSIN-β, but not in cells transfected with the pCMV-β (Figure 2A), indicating the presence of both sense and anti-sense RNA of the nsp4 gene in cells transfected with the pSIN-β. The production of dsRNA in cells transfected with the pSIN-β was further confirmed using ELISA. As shown in Figure 2B, TC-1 cells transfected with pSIN-β had an elevated level of dsRNA compared to untransfected cells, whereas cells transfected with pCMV-β and the untransfected cells had a similar level of dsRNA. The dsRNA generated within cells transfected with the pSIN-β seemed to be functional because the number of live cells in samples transfected with pSIN-β decreased gradually with the increase in incubation time, in contrast to the increase in the number of live cells in samples transfected with the pCMV-β (Figure 2C). As expected, the pSIN-β-Δnsp no longer caused a decrease in the number of live cells when transfected into the TC-1 tumor cells (Figure 2C), indicating the significance of the nsp1-4 genes for the pSIN-β plasmid to be functional. This observation is in agreement with the finding by Leitner *et al*. (2004), who showed that the survival of BHK-21 cells transfected with a replicase-based plasmid was significantly lower than cells transfected with a conventional CMV promoter-driven plasmid [40]. The cell death after transfection with the pSIN-β was likely caused by the pro-apoptotic dsRNA produced by the sindbis RNA replicase complex [40]

### Treatment of tumor-bearing mice with pSIN-β plasmid caused tumor regression

**Figure 3:**
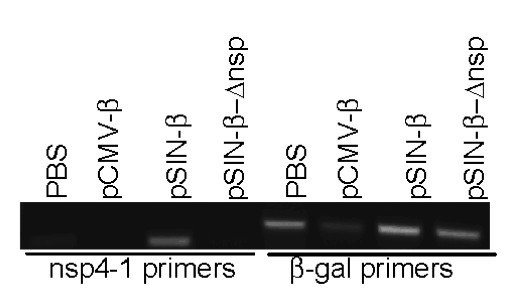
***In vivo *expression of nsp4 gene**. Twenty-four h after i.m. injection with PBS, pCMV-β, pSIN-β, or pSIN-β-Δnsp, total RNA was extracted from the muscle tissues and RT-PCR-amplified to detect the expression of nsp4 and β-gal genes.

Prior to carrying out tumor treatment studies, the ability of the pSIN-β to express the nsp1-4 genes *in vivo *was examined. As shown in Figure 3 using RT-PCR, nsp4 RNA expression was detected only in mouse muscle tissues injected with the pSIN-β, not in the ones injected with the pCMV-β or the pSIN-β-Δnsp, demonstrating that only the pSIN-β was capable of expressing the nsp genes *in vivo*. The β-gal mRNA was present in all the mouse muscle tissues since the β-gal gene is endogenous (Figure 3).

**Figure 4:**
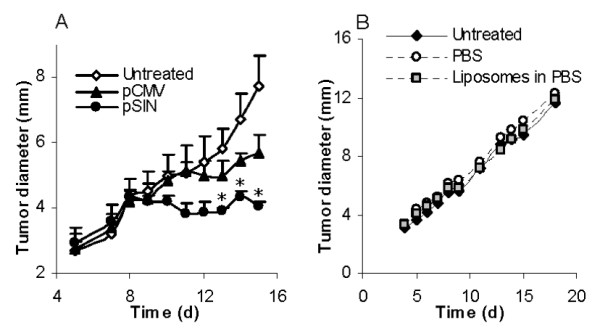
**Treatment of mice with the pSIN-β caused TC-1 tumor regression**. **(A)**. C57BL/6 mice (n = 5) were s.c. implanted with TC-1 tumor cells (5 × 10^5^) on day 0. DNA-liposome lipoplexes were injected (s.c., p.t.) for 10 consecutive days, starting on day 5 (25 μg DNA per day). (*) indicates that on days 13-15 the values of pCMV-β and pSIN-β were different from each other (p < 0.05). **(B)**. Peritumoral injection of liposomes alone or sterile PBS did not affect the growth of the TC-1 tumors. Mice (n = 5) with TC-1 tumors were injected (p.t.) with sterile PBS or liposomes in PBS (dose equivalent to that injected in the DNA-liposome lipoplexes) for 10 consecutive days, starting on day 4. Data shown were mean ± S.E.M.

To evaluate the extent to which the pSIN-β can control the growth of tumors pre-established in mice, mouse TC-1 lung cancer cells were seeded in mice. When tumors reached 3-4 mm in diameter, mice were treated with pSIN-β or pCMV-β daily for 10 days. Mice in the negative control group were not treated. As shown in Figure 4A, TC-1 tumors grew significantly slower in mice that received the pSIN-β plasmid than in mice that received the pCMV-β plasmid. In fact, 25 days after cell seeding, only 20% or 40% of tumor-bearing mice that were left untreated or received the pCMV-β plasmid, respectively, were alive, but all mice that received the pSIN-β were still alive (Table 1). Moreover, on day 25, there was only one mouse in the group that received the pSIN-β plasmid had a tumor of 3.1 mm in diameter, which completely regressed on day 37 (Table 1). Clearly, the pSIN-β plasmid was more effective in controlling the growth of the TC-1 tumors than the pCMV-β plasmid. Finally, mice in the negative control group were left untreated because it was shown that repeated peritumoral injection of sterile PBS or the liposomes did not have any effect on the growth of the TC-1 tumors as compared to mice left untreated (Figure 4B), demonstrating that potential inflammations caused by the liposomes alone or by the peritumoral injections *per se *were not responsible for the anti-tumor activity observed in Figure 4A.

**Table 1:** Treatment with pSIN-β plasmid caused TC-1 tumor regression.

	Untreated	**pCMV-**β	**pSIN-**β
**No. of mice alive **^a^	1/5	2/5	5/5
**Tumor size (mm)**	9.1	8.9, 6.1	0, 0, 0, 0, 3.1^b^

Data shown are 25 days after tumor cell seeding into mice.

a. Shown are number of live mice/total number of mice.

b. On day 37, all mice that were treated with the pSIN-β became tumor-free.

### The anti-tumor activity from pSIN-β required functional replicase genes nsp1-4

**Figure 5:**
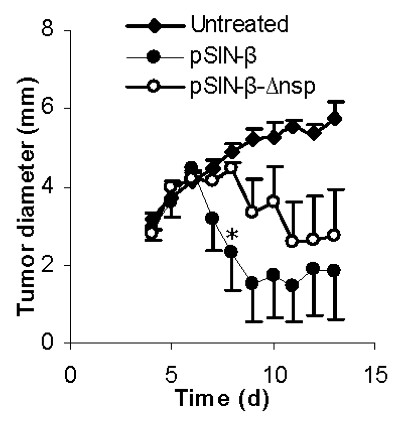
**Deletion of the replicase genes (nsp1-3 and part of nsp4) from the pSIN-β plasmid significantly decreased the anti-tumor activity of the plasmid**. C57BL/6 mice (n = 4-5) were s.c. implanted with TC-1 tumor cells (5 × 10^5^) on day 0. From days 4 to 13, mice were injected (s.c., p.t.) with lipoplexes prepared with pSIN-β (25 μg) or pSIN-β-Δnsp (25 μg). *, On day 8, p = 0.05, pSIN-β vs. pSIN-β-Δnsp.

To understand whether the sindbis replicase genes, nsp1-4, were related to the anti-tumor activity of the pSIN-β plasmid, the anti-tumor activity of the pSIN-β-Δnsp was compared to that of the pSIN-β. When used to treat the TC-1 tumors in mice, the pSIN-β-Δnsp was significantly less effective in controlling the growth of the tumors than the pSIN-β in the beginning (Figure 5), demonstrating that the nsp1-4 genes, which were responsible for the dsRNA production, played a significant role in the anti-tumor activity of the pSIN-β plasmid. It needs to be noted that the TC-1 tumor cells are strongly immunogenic, and it was expected the peritumoral injection of pSIN-β-Δnsp to show anti-tumor activity because the plasmid, with CpG motifs, can activate innate anti-tumor immune responses [41].

**Figure 6:**
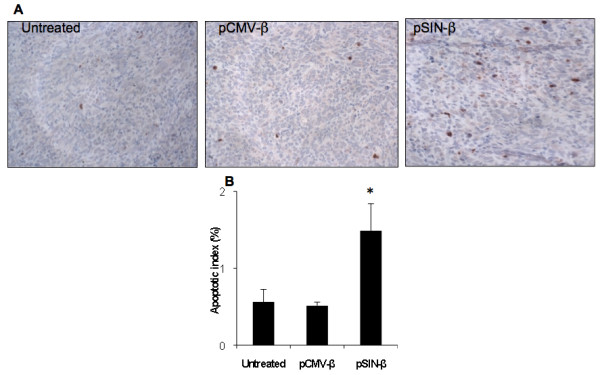
**Injection with pSIN-β promoted more tumor cells to undergo apoptosis**. **(A)**. Micrographs of tumors stained against anti-caspase-3 (brown). **(B)**. Apoptotic index. Data shown were mean ± S.E.M. The number of mice in each group was 3-4. (*) Indicates that the value of pSIN-β differed from that of the others (ANOVA, p = 0.03).

**Figure 7:**
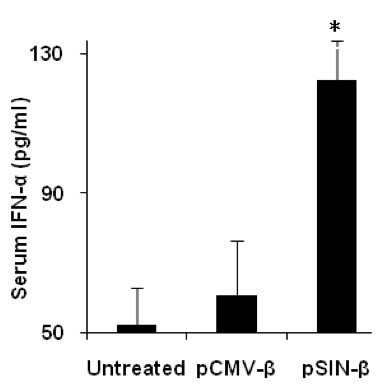
**The pSIN-β plasmid induced IFN-α production in mouse sera**. IFN-α levels in blood were measured 10 h after injection (n = 4). Data reported are means ± SEM. (*, p < 0.05, pCMV-β vs. pSIN-β).

As shown in Figure 6 more cells in tumors treated with the pSIN-β plasmid underwent apoptosis than in tumors treated with the pCMV-β plasmid. We suspect that the increased apoptosis in tumors that received the pSIN-β plasmid was related to the plasmid's ability to produce dsRNA in transfected cells. However, it is unclear to what extent the apoptosis was caused directly by dsRNA produced by the pSIN-β plasmid. Double stranded RNA is pro-apoptotic [2], but the type I IFNs induced by dsRNA are pro-apoptotic as well [2]. Moreover, the unmethylated CpG motifs on the plasmid, the dsRNA *per se*, and type I IFN are all known to be able to activate innate immunity such as natural killer (NK) cells, which can cause tumor death [42]. In fact, it was shown that subcutaneous injection of the pSIN-β plasmid induced an elevated level of IFN-α in mouse serum samples (Figure 7).

### Adaptive immunity contributed to the anti-tumor activity from pSIN-β

**Figure 8:**
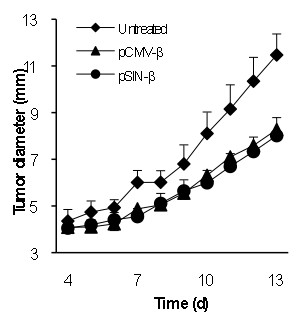
**The pSIN-β plasmid was no longer more effective than pCMV-β against tumors in athymic mice**. Mice (n = 6-8) were s.c. implanted with TC-1 tumor cells (5 × 10^5^) on day 0. From days 4 to 13, mice were injected (s.c., p.t) with lipoplexes prepared with pSIN-β (25 μg) or pCMV-β (25 μg).

TC-1 tumor cells are highly immunogenic in C57BL/6 mice due to the human papillomavirus E6 an E7 genes in the TC-1 cells [43]. Data from several recent studies have shown that tumor cells with intracellular dsRNA were more immunogenic than tumor cells physically mixed with dsRNA [19-22]. Therefore, it was expected that adaptive immune responses have contributed, to a certain extent, to the anti-tumor activity from the pSIN-β plasmid. To test this hypothesis, the same TC-1 tumors established in athymic mice were treated with pSIN-β or pCMV-β. As shown in Figure 8 the pSIN-β was no longer more effective than the pCMV-β in controlling the growth of the TC-1 cells, indicating that adaptive immunity contributed to the anti-tumor activity from the pSIN-β plasmid. However, the adaptive immunity was not absolutely required for the pSIN-β to have anti-tumor activity because recent preliminary data in our lab showed that in athymic mice, the pSIN-β caused total regression of pre-established model human tumors when targeted into the tumor cells using a tumor-specific ligand (Rodriguez and Cui, unpublished data).

### Unmethylated CpG motifs contributed to the anti-tumor activity of the pCMV-β

**Figure 9:**
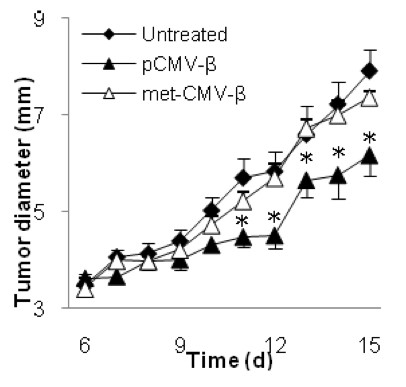
**Unmethylated CpG motifs contributed to the anti-tumor activity of the pCMV-β**. C57BL/6 mice (n = 5-6) were implanted with TC-1 tumor cells (5 × 10^5^) on day 0. From days 6 to 15, mice were injected (s.c., p.t.) with lipoplexes prepared with unmethylated or methylated pCMV-β (pCMV-β or met-CMV-β, 25 μg). Data shown were mean ± S.E.M. (*) indicates that on days 11 to 15, the values of pCMV-β and met-CMV-β were different from each other (p < 0.05).

The anti-tumor effect from the pCMV-β plasmid was likely due to the unmethylated CpG motifs present on the plasmid [41]. As shown in Figure 9 methylation of the pCMV-β depleted the plasmid's ability to inhibit the growth of the TC-1 tumor cells in mice. This is in agreement with a previous report showing that plasmid DNA itself had anti-tumor activity because the unmethylated CpG motifs on the plasmid can activate innate immunity [41]. Therefore, it is possible that both dsRNA produced by the RNA replicase complex encoded by the nsp1-4 genes and the unmethylated CpG motifs on the pSIN-β plasmid may have contributed to the anti-tumor activity from the pSIN-β.

### The pSIN-β plasmid was effective against B16 melanoma in mice as well

**Figure 10:**
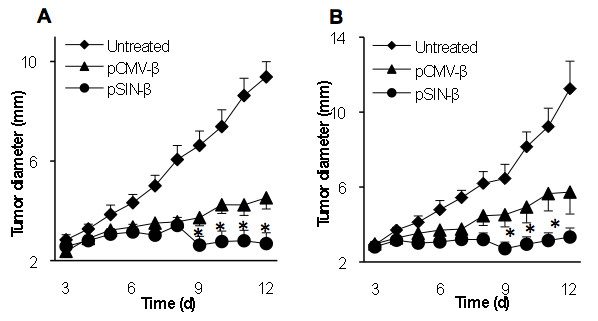
**pSIN-β was more effective than pCMV-β in controlling the growth of mouse B16-F10 and B16-OVA melanomas as well**. C57BL/6 mice (n = 6-7) were implanted with B16-F10 **(A) **or B16-OVA **(B) **cells on day 0. DNA-liposome lipoplexes were injected (s.c., p.t.) for 10 consecutive days starting on day 3 (25 μg DNA per day). (*) indicate that on days 9-12 for B16-F10 (or days 9-11 for B16-OVA), the values of pCMV-β and pSIN-β were different from each other (p < 0.05). Data shown are mean ± S.E.M.

To test whether the pSIN-β was effective against tumors other than the TC-1, mice with pre-established B16-F10 or B16-OVA tumors were treated similarly. As shown in Figure 10 the pSIN-β plasmid significantly controlled the growth of both poorly immunogenic B16-F10 tumors (Figure 10A) and the more immunogenic B16-OVA tumors (Figure 10B), indicating that the approach of controlling tumor growth with the replicase-based plasmid was not limited to the TC-1 tumors and likely not limited to highly immunogenic tumors as well. Again, it is not surprising that the pCMV-β also showed anti-tumor activity against the B16 melanoma. In a previous study, McCray *et al*. (2006) showed that intratumoral injection of an empty pcDNA3.1 delayed the growth of B16 tumors, as compared to the injection of saline [44]. It was also shown that intratumoral injection of the pcDNA3.1 followed by *in vivo *electroporation further improved the anti-tumor activity [44]. In the present study, the repeated peritumoral injection of the pCMV-β complexed with cationic liposomes may have improved the non-specific anti-tumor activity from the plasmid.

## Conclusions

A RNA replicase-based plasmid that did not encode any relevant functional gene was showed to have anti-tumor activity. The anti-tumor activity of the RNA replicase-encoding plasmid was likely due to its ability to allow the transfected tumor cells to produce dsRNA and to activate innate and adaptive immunity. In the present study, for proof-of-concept purpose, the RNA replicase encoding plasmid was dosed to mice by subcutaneous peritumoral injection. Although feasible for tumors such as head and neck cancers, certain non-metastasized melanomas, and brain tumors, peritumoral or intratumoral injection is expected to be difficult to operate for many other solid tumors. We are in the process of developing a liposome-based system to target the RNA replicase encoding plasmid into tumor cells by the intravenous route. Treatment of poorly immunogenic tumors such as B16-F10 melanoma in animal models is a good simulation of conditions observed in cancer patients [45], and the data in the present study showed that both highly immunogenic and poorly immunogenic solid tumors were receptive to treatment with a RNA replicase based plasmid. Our results suggested a novel approach to cancer molecular therapy.

## Authors' contributions

BLR, Figure 1, 2A, 2C, 3, 4, 5, 6, 7, 8, 9, 10, experimental design and manuscript preparation; YZ, Figure 2B; WC, Figure 2C and assistance in animal experiments and design; RW, construction of pSIN-β and manuscript review; ZC research design and manuscript preparation. All authors read and approve the final manuscript.

## Declaration of competing interests

The authors declare that they have no competing interests.

## Pre-publication history

The pre-publication history for this paper can be accessed here:

http://www.biomedcentral.com/1471-2407/11/110/prepub
